# Association of Klotho Protein Levels and *KL-VS* Heterozygosity With Alzheimer Disease and Amyloid and Tau Burden

**DOI:** 10.1001/jamanetworkopen.2022.43232

**Published:** 2022-11-22

**Authors:** Gøril Rolfseng Grøntvedt, Sigrid Botne Sando, Camilla Lauridsen, Geir Bråthen, Linda R. White, Øyvind Salvesen, Dag Aarsland, Erik Hessen, Tormod Fladby, Knut Waterloo, Katja Scheffler

**Affiliations:** 1 Department of Neurology and Clinical Neurophysiology, University Hospital of Trondheim, Trondheim, Norway; 2 Department of Neuromedicine and Movement Science, Faculty of Medicine and Health Sciences, Norwegian University of Science and Technology, Trondheim, Norway; 3 KG Jebsen Centre for Alzheimer’s Disease, Kavli Institute for Systems Neuroscience, Trondheim, Norway; 4 Unit for Applied Clinical Research, Faculty of Medicine and Health Sciences, Norwegian University of Science and Technology, Trondheim, Norway; 5 Centre for Age-Related Medicine, Stavanger University Hospital, Stavanger, Norway; 6 Department of Old Age Psychiatry, Institute of Psychiatry, Psychology and Neuroscience, King’s College London, London, United Kingdom; 7 Department of Psychology, University of Oslo, Oslo, Norway; 8 Department of Neurology, Akershus University Hospital, Lørenskog, Norway; 9 Institute of Clinical Medicine, Campus Ahus, University of Oslo, Oslo, Norway; 10 Department of Neurology, University Hospital of North Norway, Tromsø, Norway

## Abstract

**Importance:**

Identification of proteins and genetic factors that reduce Alzheimer disease (AD) pathology is of importance when searching for novel AD treatments. Heterozygosity of the *KL-VS* haplotype has been associated with reduced amyloid and tau burden. Whether this association is mediated by the Klotho protein remains unclear.

**Objectives:**

To assess concentrations of Klotho in cerebrospinal fluid (CSF) and plasma among cognitively healthy controls and patients with AD and to correlate these findings with *KL-VS* heterozygosity status and amyloid and tau burden.

**Design, Setting, and Participants:**

This case-control study combined 2 independent case-control AD cohorts consisting of 243 referred patients with AD and volunteer controls recruited from January 1, 2009, to December 31, 2018. Klotho levels were measured in CSF and plasma and correlated with *KL-VS* heterozygosity status and levels of CSF amyloid-β 42 (Aβ42), total tau, and phosphorylated tau. Statistical analysis was performed from January 1, 2021, to March 1, 2022.

**Main Outcomes and Measures:**

Associations of Klotho levels in CSF and plasma with levels of CSF biomarkers were analyzed using linear regression. Association analyses were stratified separately by clinical groups, *APOE4* status, and *KL-VS* heterozygosity*.* Pearson correlation was used to assess the correlation between CSF and plasma Klotho levels.

**Results:**

A total of 243 participants were included: 117 controls (45 men [38.5%]; median age, 65 years [range, 41-84 years]), 102 patients with mild cognitive impairment due to AD (AD-MCI; 59 men [57.8%]; median age, 66 years [range, 46-80 years]), and 24 patients with dementia due to AD (AD-dementia; 12 men [50.0%]; median age, 64.5 years [range, 54-75 years]). Median CSF Klotho levels were higher in controls (1236.4 pg/mL [range, 20.4-1726.3 pg/mL]; β = 0.103; 95% CI, 0.023-0.183; *P* = .01) and patients with AD-MCI (1188.1 pg/mL [range, 756.3-1810.3 pg/mL]; β = 0.095; 95% CI, 0.018-0.172; *P* = .02) compared with patients with AD-dementia (1073.3 pg/mL [range, 698.2-1661.4 pg/mL]). Higher levels of CSF Klotho were associated with lower CSF Aβ42 burden (β = 0.519; 95% CI, 0.201-0.836; *P* < .001) and tau burden (CSF total tau levels: β = −0.884; 95% CI, 0.223 to −0.395; *P* < .001; CSF phosphorylated tau levels: β = −0.672; 95% CI, −1.022 to −0.321; *P* < .001) independent of clinical, *KL-VS* heterozygosity, or *APOE4* status. There was a weak correlation between Klotho CSF and plasma levels among the entire cohort (Pearson correlation *r* = 0.377; *P* < .001).

**Conclusions and Relevance:**

The findings of this case-control study suggest that Klotho protein levels were associated with clinical stages of AD, cognitive decline, and amyloid and tau burden and that these outcomes were more clearly mediated by the protein directly rather than the *KL-VS* heterozygosity variant. When selecting individuals at risk for clinical trials, the Klotho protein level and not only the genetic profile should be considered.

## Key Points

**Question:**

What is the association between Klotho protein levels in cerebrospinal fluid (CSF) and plasma, heterozygosity status of the *KL-VS* haplotype, and amyloid and tau burden among cognitively healthy controls and patients with Alzheimer disease (AD)?

**Findings:**

In this cross-sectional study of 243 patients with AD and controls, CSF Klotho levels were significantly higher among controls and individuals with mild cognitive impairment due to AD compared with individuals with dementia due to AD. Furthermore, CSF Klotho levels were significantly associated with lower levels of CSF AD core biomarkers independent of clinical, *KL-VS* heterozygosity, or *APOE4* status.

**Meaning:**

These findings suggest that Klotho protein levels, but not *KL-VS* heterozygosity status, differ in clinical stages of AD and are associated with amyloid and tau burden.

## Teaser

This case-control study assesses concentrations of Klotho protein in cerebrospinal fluid and plasma among cognitively healthy controls and patients with Alzheimer disease (AD) and correlates these findings with *KL-VS* heterozygosity status and amyloid and tau burden.

## Introduction

Klotho is a transmembrane glycoprotein expressed mainly in the kidney and choroid plexus of the brain. The function of Klotho in the brain was initially derived from *klotho* (OMIM 604824) knockout mice that demonstrated cognitive impairment, premature death, and synaptic loss.^1,2^ Conversely, overexpression of *klotho* extends the lifespan, improves cognitive function, and reduces age-associated phenotypes in mice.^3,4^ A common haplotype consisting of 6 missense variants in the human *Klotho* gene is termed *KL-VS*.^5^ Heterozygosity for *KL-VS* is associated with higher levels of Klotho, longevity, greater cortical volume, and better global cognition in older adults, suggesting a possible protective role of Klotho in normal aging.^6,7,8,9^ Recent studies have shown an association between *KL-VS* heterozygosity status and reduced amyloid burden and risk of Alzheimer disease (AD) among people carrying the *APOE4* allele.^10,11,12^ Moreover, *KL-VS* heterozygosity has been associated with lower amyloid-related tau pathology among elderly individuals at risk for AD^13^ and with a possible protective effect against an age-related increase in tau burden.^14^

To our knowledge, few studies have measured concentrations of Klotho in patients with AD. In cerebrospinal fluid (CSF), Klotho levels decreased with age and were higher in healthy elderly individuals compared with patients with AD.^15^ Low plasma Klotho levels have been associated with lower Mini-Mental State Examination (MMSE)^16^ scores and an increased risk of vascular dementia, but not with late-onset AD.^17^ No study has so far correlated Klotho levels with the *KL-VS* haplotype in patients with AD, to our knowledge.

The aim of this study was to assess concentrations of Klotho in CSF and plasma and to correlate these findings with *KL-VS* heterozygosity status to explore a possible association with amyloid and tau burden among a control group of cognitively healthy elderly individuals and patients with AD.

## Methods

### Participants

In total, 243 participants were selected from 2 Norwegian cohorts from January 1, 2009, to December 31, 2018; demographic and associated data are shown in Table 1. Controls (n = 54) and patients with mild cognitive impairment due to AD (AD-MCI; n = 30) or dementia due to AD (AD-dementia) (n = 19), defined according to National Institute on Aging–Alzheimer’s Association (NIA-AA) criteria,^18,19^ were recruited from the Department of Neurology, St Olavs Hospital, Trondheim, Norway (Trønderbrain cohort).^20^ Controls (n = 63) and participants with AD-MCI (n = 72) or AD-dementia (n = 5), defined according to NIA-AA criteria,^18,19^ were recruited from the Norwegian multicenter Dementia Disease Initiation (DDI) study.^21^ Exclusion criteria for both cohorts were applied as described elsewhere.^20,21^ All patients had pathologic concentrations of amyloid-β 42 (Aβ42) in CSF, and their disease was considered part of the AD continuum.^18,19,22^ The cutoff for Aβ42 in CSF in the Trønderbrain cohort was less than 630 pg/mL, calculated by maximizing the Youden index.^23^ The cutoff for Aβ42 in CSF in the DDI study was less than 708 pg/mL, determined by a positron emission tomographic [^18^F]-flutemetamol uptake study based on the same cohort.^24^ Controls were assessed as being cognitively healthy without signs of any neurologic disorders and with normal concentrations of Aβ42 in CSF. Written informed consent was obtained from all patients or suitable proxies, as well as from all control participants. The study was approved by the Regional Committees for Medical Research Ethics in Central and South-East Norway. This case-control study is reported following the Strengthening the Reporting of Observational Studies in Epidemiology (STROBE) reporting guideline.

**Table 1.** Demographic Data of the Clinical Groups in the DDI Study and Trønderbrain Cohorts

Characteristic	Patients, No. (%)			
	Control group	AD group	AD-MCI group	AD-dementia group
**DDI study cohort**				
No.	63	77	72	5
Age, median (range), y	62 (41-77)	68 (46-80)^a^	68 (46-80)^a^	73 (58-75)
Sex				
Male	27 (42.9)	47 (61.0)^a^	44 (61.1)^a^	3 (60.0)
Female	36 (57.1)	30 (39.0)^a^	28 (38.9)^a^	2 (40.0)
*APOE4* allele	25 (39.7)	56 (72.7)^a^	53 (73.6)^a^	3 (60.0)
MMSE score, median (range)	30 (27-30)	27 (18-30)^a^	27 (21-30)^a^	25 (18-28)^a^
**Trønderbrain cohort**				
No.	54	49	30	19
Age, median (range), y	68 (57-84)	64 (54-72)^a^	64 (54-72)^a^	64 (54-69)^a^
Sex				
Male	18 (33.3)	24 (49.0)	15 (50.0)	9 (47.4)
Female	36 (66.7)	25 (51.0)	15 (50.0)	10 (52.6)
*APOE*4 allele	20 (37.0)	42 (85.7)	24 (80.0)^a^	18 (94.7)^a^
MMSE score, median (range)	30 (28-30)	26 (16-30)^a^	28 (23-30)^a^	24 (16-27)^a^^,^^b^

Abbreviations: AD, Alzheimer disease; AD-MCI, AD with mild cognitive impairment; DDI, Dementia Disease Initiation; MMSE, Mini-Mental State Examination.

^a^ *P* < .01 compared with control group.

^b^ *P* < .01 compared with AD-MCI group.

Neurologic examination, blood screening, CSF collection, and cognitive tests, including the MMSE, were performed for all participants. Genotyping of *APOE* was performed as described elsewhere.^20,21,25^

### Assessment of *KL-VS* Heterozygosity

Genotyping was conducted at deCODE Genetics (Reykjavik, Iceland). DNA was extracted from whole blood and genotyped with the Human Omni Express-24 version 1.1 (Illumina Inc) in accordance with the standard Illumina protocol, as described elsewhere.^26^ A total of 20 participants were excluded from the study cohort because of *KL-VS* homozygosity.

### Sampling and Analysis of CSF and Plasma

Samples of CSF and plasma were collected from all participants following procedures described elsewhere.^20,27^ Commercial enzyme-linked immunosorbent assays (ELISAs; Fujirebio) were used to measure CSF levels of Aβ42, total tau (T-tau), and phosphorylated tau (P-tau). All samples were analyzed at Akershus University Hospital, Lørenskog, Norway, except those from the controls in Trønderbrain, which were analyzed at the neurobiological laboratory in Trondheim, Norway. Because of minor differences in the measurements between the 2 sites, control samples in Trønderbrain were excluded from the correlation analyses between CSF and plasma Klotho levels and levels of core CSF biomarkers.

### Analysis of Klotho Concentrations

Klotho levels were analyzed in CSF and plasma samples using the commercially available human soluble α-Klotho Assay ELISA kit (Immuno-Biological Laboratories Co Ltd). Plasma samples were diluted 1:2, whereas CSF samples were analyzed undiluted. Klotho measurements in plasma were missing for 12 participants (controls, 11; AD-MCI group, 1) and Klotho measurements in CSF were missing for 9 control participants, as samples had been exhausted.

### Statistical Analysis

Statistical analyses were performed from January 1, 2021, to March 1, 2022, using SPSS, version 28 (IBM Corp). Normality was assessed through the inspection of Q-Q plots, histograms, and the Shapiro-Wilks test of normality. Cerebrospinal fluid AD biomarkers had nonnormal distributions, and group comparisons were performed using the Mann-Whitney and Kruskal-Wallis tests. Group comparisons of CSF and plasma Klotho levels were performed by log-transforming Klotho levels, applying multivariable linear regression adjusting for age, sex, and *APOE4* and *KL-VS* heterozygosity status*.* The association of age and sex with log Klotho levels was examined separately by univariable linear regression. Associations between CSF Klotho levels and plasma Klotho levels with levels of core CSF AD biomarkers were analyzed using univariable linear regression and stratified separately by clinical groups, *APOE4* status, and *KL-VS* heterozygosity*.* Pearson correlation was used to assess the correlation between log levels of CSF Klotho and plasma Klotho. Analyses and associations were considered significant at a 2-sided *P* < .05.

## Results

The demographic and clinical characteristics of the participants in this case-control study across 2 AD cohorts are summarized in Table 1. A total of 243 participants were included: 117 controls (45 men [38.5%] and 72 women [61.5%]; median age, 65 years [range, 41-84 years]), 102 patients with AD-MCI (59 men [57.8%] and 43 women [42.2%]; median age, 66 years [range, 46-80 years]), and 24 patients with AD-dementia (12 men [50.0%] and 12 women [50.0%]; median age, 64.5 years [range, 54-75 years]).

### Concentrations of CSF Klotho in Clinical Groups

**Figure 1.**
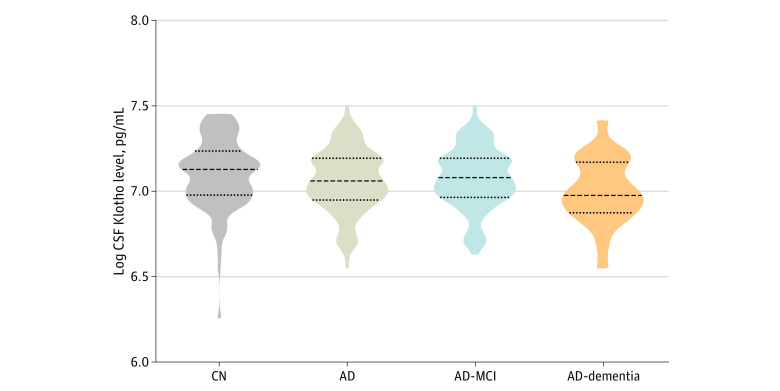
Cerebrospinal Fluid (CSF) Klotho Levels in the Clinical Groups This violin plot shows concentrations of Klotho in CSF measured by commercially available human soluble α-Klotho assay enzyme-linked immunosorbent assy kit in different clinical groups. AD indicates Alzheimer disease; AD-MCI, AD with mild cognitive impairment; and CN, cognitively healthy controls.

Figure 1 shows CSF levels of Klotho in the clinical groups. The control group had the highest median CSF Klotho concentration vs the total AD group (1236.4 pg/mL [range, 20.4-1726.3 pg/mL] vs 1162.6 pg/mL [range, 698.2-1810.3 pg/mL]). Within the AD group, the median concentration of Klotho in CSF decreased with disease severity, from 1188.1 pg/mL (range, 756.3-1810.3 pg/mL) in the AD-MCI group to 1073.3 pg/mL (range, 698.2-1661.4 pg/mL) in the AD-dementia group. The median concentration of CSF Klotho was significantly higher in the control group (β = 0.103; 95% CI, 0.023-0.183; *P* = .01) and AD-MCI group (β = 0.095; 95% CI, 0.018-0.172; *P* = .02) compared with the AD-dementia group, using multivariable linear regression adjusting for age, sex, *KL-VS* heterozygosity, and *APOE4* status. There were no significant differences in median CSF Klotho concentrations between the control group and the AD-MCI group or between the control group and the entire AD group.

### Concentrations of CSF Klotho and Age

**Figure 2.**
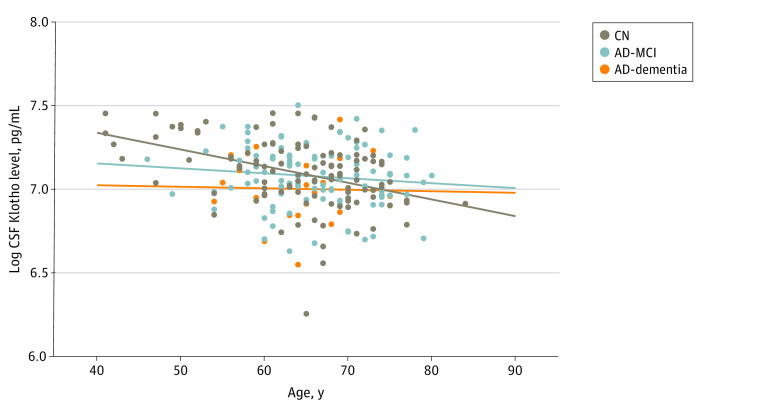
Scatterplot of Cerebrospinal Fluid (CSF) Klotho Levels and Age in the Clinical Groups This scatterplot shows logarithmic-transformed concentrations of Klotho in CSF and age in different clinical groups. AD-MCI indicates Alzheimer disease with mild cognitive impairment; and CN, cognitively healthy controls.

Analysis showed that CSF Klotho levels were weakly, but significantly, correlated with age in the entire cohort (*R*^2^ = 0.070; *P* < .001). When stratifying the analysis by sex, the result remained significant (women, *R*^2^ = 0.098; *P* < .001; men, *R*^2^ = 0.039; *P* = .04). The correlation significantly differed between the clinical groups and was associated with the control group (*R*^2^ = 0.168; *P* < .001) but not the other groups (Figure 2). Analysis showed significantly lower median CSF Klotho levels in the AD-dementia group compared with the control group among participants younger than 65 years (AD-dementia, 1057 pg/mL [range, 698.18-1411.9 pg/mL]; controls, 1313.1 pg/mL [range 846.5-1726.3 pg/mL]; *P* = .003). The same was not found in the older group (AD-dementia group: median, 1097.5 pg/mL [range, 888.7-1661.4 pg/mL]; controls: median, 1154 pg/mL [range, 520.4-1684.7 pg/mL]; *P* = .71).

### Correlations of CSF Klotho Levels and MMSE Score

There was a weak but significantly positive association between MMSE score and CSF Klotho levels in the entire cohort (*R*^2^ = 0.025; *P* = .02). Subgroup analysis showed that CSF Klotho levels were significantly associated with MMSE score in the entire AD group (*R*^2^ = 0.048; *P* = .01) but not the control, AD-MCI, and AD-dementia groups.

### Concentrations of CSF Klotho Stratified by *APOE4* Status and Sex

There was no significant difference in CSF Klotho concentrations stratified by *APOE4* status in the entire study population, the controls, or the combined AD-MCI and AD-dementia groups. Individuals in the AD-dementia group carrying *APOE4* had significantly lower median concentrations of Klotho than noncarriers (*APOE4* carriers, 1050.8 pg/mL [range, 698.1-1661.4 pg/mL]; noncarriers, 1378.6 pg/mL [range, 1357.7-1411.0]; *P* < .001). This result might be due to low sample size, as only 4 of 25 patients in this group were *APOE4* noncarriers. There were no significant differences in concentrations of Klotho in CSF based on sex in the study population or at any group level.

### Association Between CSF Klotho Levels and Levels of CSF AD Biomarkers

**Figure 3.**
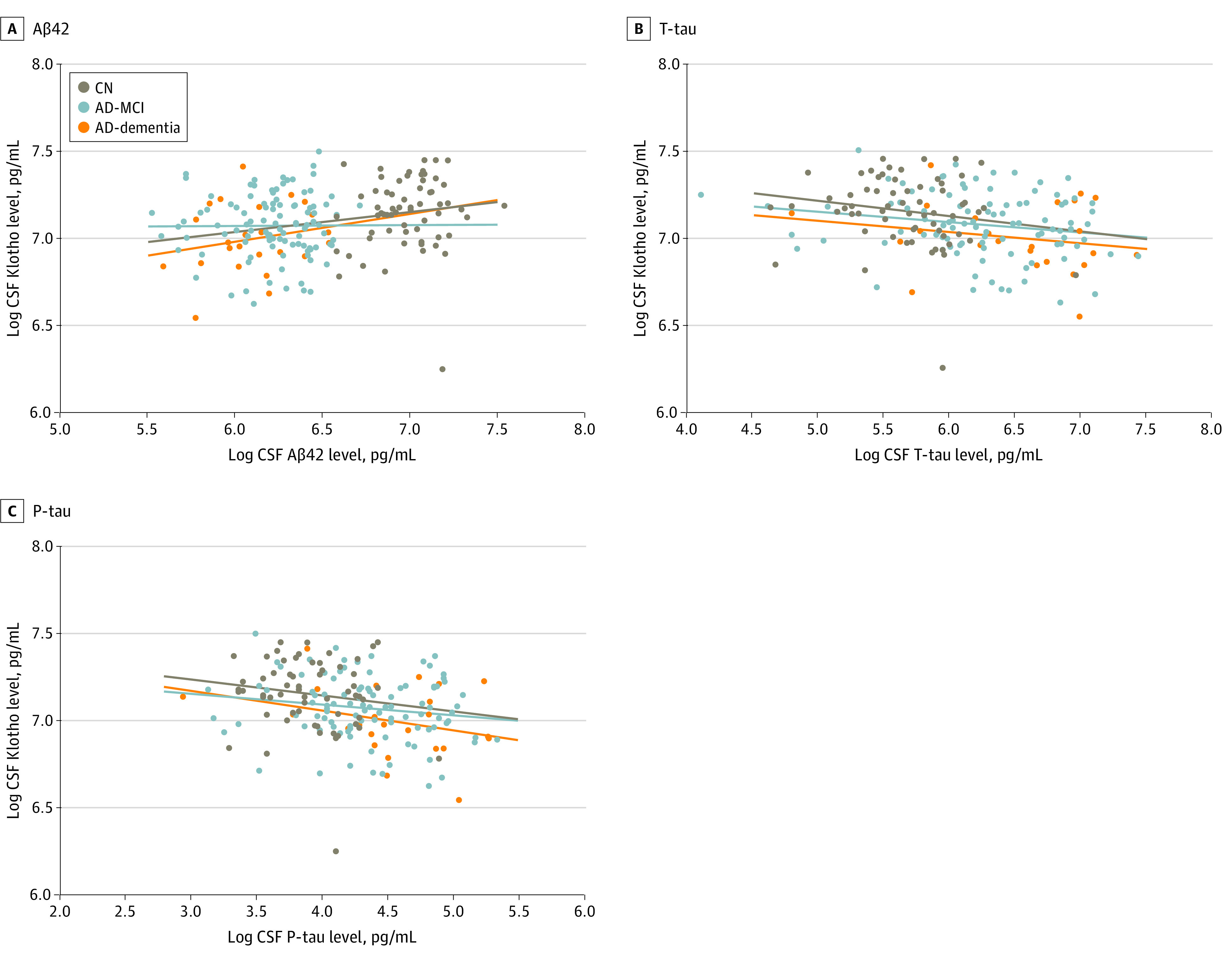
Scatterplot of Cerebrospinal Fluid (CSF) Klotho and Core Alzheimer Disease (AD) Biomarkers in CSF at Baseline in the Clinical Groups This scatterplot shows logarithmic-transformed concentrations of Klotho in CSF and core CSF AD biomarkers in different clinical groups. Aβ42 indicates amyloid-β 42; AD-MCI, AD with mild cognitive impairment; CN, cognitively healthy controls; P-tau, phosphorylated tau; and T-tau, total tau.

The concentrations of Aβ42, T-tau, and P-tau in CSF in the clinical groups in the DDI study cohort and the Trønderbrain cohort are shown in Table 2. Because of differences in site procedures, the control group from Trønderbrain (n = 54) had to be removed from the analysis. The associations between CSF Klotho levels and levels of core CSF AD biomarkers in the clinical groups are shown in Figure 3. A significant association was found for all 3 CSF AD biomarkers in the entire cohort; CSF Klotho levels had a significant positive association with CSF Aβ42 levels (β = 0.519; 95% CI, 0.201-0.836; *P* < .001), a significant negative association with CSF T-tau levels (β = −0.884; 95% CI, 0.223 to −0.395; *P* < .001), and a significant negative association with CSF P-tau levels (β = −0.672; 95% CI, −1.022 to −0.321; *P* < .001). Neither stratifying the analysis by clinical stage nor based on *APOE4* status and *KL-VS* heterozygosity status showed a significant difference in the associations between CSF Klotho levels and levels of any of the 3 AD CSF biomarkers (eTables 1 and 2 in the Supplement).

**Table 2.** Concentrations of Core AD Biomarkers in CSF at Baseline in the Clinical Groups

Characteristic	Median (range), pg/mL			
	Control group	AD group	AD-MCI group	AD-dementia group
**DDI study cohort**				
No.	63	77	72	5
CSF				
Aβ42	1090.0 (720.0-1880.0)	535.0 (250.0-706.0)^a^	537.5 (250.0-706.0)^a^	413.0 (370.0-600.0)^a^
T-tau	294.0 (102.0-1050.0)	520.0 (60.0-1670.0)^a^	502.0 (60.0-1370.0)^a^	1040.0 (507.0-1670.0)^a^^,^^b^
P-tau	48.0 (27.0-134.0)	75.7 (23.0-196.0)^a^	74.0 (23.0-177.0)^a^	134.0 (67.0-196.0)^a^
**Trønderbrain cohort**				
No.	54	49	30	19
CSF				
Aβ42	1054.6 (665.8-1674.1)	447.4 (173.0-611.6)^a^	433.0 (173.0-602.6)^a^	476.8 (322.1-611.6)^a^
T-tau	272.6 (137.5-665.9)	515.5 (135.4-2325.3)^a^	438.4 (135.4-2325.3)^a^	624.2 (221.9-1391.0)^a^
P-tau	51.9 (32.8-86.4)	76.4 (15.9-168.8)^a^	71.9 (13.9-168.8)^a^	80.4 (35.9-156.9)^a^

Abbreviations: Aβ42, amyloid-β 42; AD, Alzheimer disease; AD-MCI, AD with mild cognitive impairment; CSF, cerebrospinal fluid; DDI, Dementia Disease Initiation; P-tau, phosphorylated tau; T-tau, total tau.

^a^ *P* < .01 compared with control group.

^b^ *P* < .05 compared with AD-MCI group.

### Concentrations of CSF Klotho Stratified by *KL-VS* Heterozygosity

There were no significant differences in the distribution of individuals with *KL-VS* heterozygosity among the clinical groups (control group, 20.5% [24 of 117]; AD-MCI group, 28.4% [29 of 102]; AD-dementia group, 29.2% [7 of 24]). Median concentrations of Klotho in CSF were significantly higher among individuals carrying *KL-VS* heterozygosity compared with noncarriers in the AD group (*KL-VS* heterozygosity carriers, 1365.9 pg/mL [range, 935.8-1810.3 pg/mL]; noncarriers, 1119.1 pg/mL [range, 698.2-1592.7]; *P* < .001), the AD-MCI group (*KL-VS* heterozygosity carriers, 1401.1 pg/mL [range, 1064.1-1810.3]; noncarriers, 1127.5 pg/mL [range, 756.2-1592.7 pg/mL]; *P* < .001), and the control group (*KL-VS* heterozygosity carriers, 1312.9 pg/mL [range, 995.1-1726.2 pg/mL]; noncarriers, 1186.9 pg/mL [range, 520.4-1723.3 pg/mL]; *P* = .02), but not in the AD-dementia group. Noncarriers of *KL-VS* heterozygosity in the control group showed significantly higher median CSF Klotho levels compared with the AD group (control group, 1186.9 pg/mL [range, 520.4-1723.3 pg/mL]; AD group, 1119.1 pg/mL [range, 698.2-1592.7 pg/mL]; *P* = .01), whereas no significant difference was found among *KL-VS* heterozygosity carriers (eFigure 1 in the Supplement). Further analysis did not show any significant differences in CSF Klotho levels among *KL-VS* heterozygosity carriers in clinical subgroups. There was a significant association between *KL-VS* heterozygosity and CSF Klotho concentrations in the entire cohort (β = 0.157; 95% CI, 0.097-0.217; *P* < .001), but independent of clinical group and *APOE4* status.

### Associations Between *KL-VS* Heterozygosity and Concentrations of Amyloid and Tau in CSF

No significant associations were found between *KL-VS* heterozygosity and levels of any of the 3 CSF AD biomarkers in the entire cohort (Aβ42: β = 0.114; 95% CI, −0.258 to 0.030; *P* = .12; T-tau: β = −0.044; 95% CI, −0.248 to 0.160; *P* = .67; P-tau: β = 0.360; 95% CI, −0.127 to 0.199; *P* = .68) or when stratified by clinical groups or *APOE4* status.

### Correlations Between CSF Klotho and Plasma Klotho Concentrations

There was a significant but weak positive correlation between Klotho concentrations in CSF and plasma in the entire cohort (Pearson correlation *r* = 0.377; *P* < .001). Stratifying the result by clinical group showed significant correlations among controls (Pearson correlation *r* = 0.336; *P* < .001) and the AD group (Pearson correlation *r* = 0.427; *P* < .001). The positive correlation in the AD group was associated mainly with the AD-MCI group (Pearson correlation *r* = 0.465; *P* < .001) and was not found in the AD-dementia group (Pearson correlation *r* = 0.213; *P* = .32) (eFigure 2 in the Supplement).

### Concentrations of Klotho in Plasma Based on *APOE4* Status, Sex, and Age in the Clinical Groups

No significant differences were found in the concentrations of plasma Klotho between the clinical groups, nor when stratifying by *APOE4* status and sex. Median plasma Klotho levels were significantly higher in men than women in the AD-dementia group (men, 871.4 pg/mL [range, 568.7-1027.9 pg/mL]; women, 711.9 pg/mL [range, 568.7-1027.9 pg/mL]; *P* = .04) but not in the other clinical groups. A significant negative association of age with plasma Klotho concentrations was seen in the control group (negative correlation, *R*^2^ = 0.147; *P* < .001) but not in other clinical groups (eFigure 3 in the Supplement).

### Associations Between Plasma Klotho Levels and Levels of Core CSF AD Biomarkers

There was a significant negative association between plasma Klotho levels and CSF T-tau levels (β = −0.398; 95% CI, −0.719 to −0.077; *P* = .02) and CSF P-tau levels (β = −0.268; 95% CI, −0.526 to −0.010; *P* = .04), but not between plasma Klotho levels and CSF Aβ42 levels (β = 0.176; 95% CI, −0.054 to −0.406; *P* = .13). Further stratification of the analysis showed no significant differences in these associations (eTable 3 in the Supplement).

### Concentrations of Plasma Klotho Stratified by *KL-VS* Heterozygosity

Among the entire cohort, carriers of *KL-VS* heterozygosity tended to have higher median plasma Klotho levels compared with noncarriers (carriers, 889.4 pg/mL [range, 501.7-1563.5 pg/mL]; noncarriers, 796.2 pg/mL [range, 351.9-2733.8 pg/mL]; *P* = .08). Although no significant difference was found in the control group, median plasma Klotho levels in the AD group were significantly higher among *KL-VS* heterozygosity carriers compared with noncarriers (carriers, 910.9 pg/mL [range, 501.7-1563.5 pg/mL]; noncarriers, 790.1 pg/mL [range, 351.9-2733.8 pg/mL]; *P* = .002). The significant increase in median Klotho levels was associated with the AD-MCI group (carriers, 936.6 pg/mL [range, 501.7-1563.5 pg/mL]; noncarriers, 790.1 pg/mL [range, 351.9-2733.8 pg/mL]; *P* = .006) and was not present in the AD-dementia group.

## Discussion

To our knowledge, this is the first study to evaluate the interaction between the *KL-VS* heterozygosity haplotype and Klotho levels in CSF and plasma and to correlate them with amyloid and tau burden in clinical stages of AD. We found the lowest concentrations of CSF Klotho in individuals with AD-dementia. Klotho levels in CSF decreased significantly with age in the control group but not the AD-MCI or AD-dementia groups. Higher levels of CSF Klotho were associated with lower CSF amyloid and tau burden, independent of clinical, *KL-VS* heterozygosity, or *APOE4* status. The same association was not found with the *KL-VS* heterozygosity haplotype, although it resulted in higher CSF Klotho levels in the control and AD-MCI groups, but not in the AD-dementia group. There was a weak correlation between Klotho levels in CSF and plasma, and although plasma Klotho levels had a positive association with tau burden, an association with amyloid burden was not found.

Our results indicate a disease stage–dependent positive association of CSF Klotho levels. Only 1 study has addressed CSF Klotho levels in patients with AD and found a similar decrease compared with controls.^15^ How Klotho is associated with progression of AD in the human brain remains unknown, but mouse studies suggest that Klotho modulates *N*-methyl-d-aspartate receptor function and activates microglia cells to promote cognitive function.^28,29^ We found significantly higher levels of CSF Klotho in *KL-VS* heterozygosity carriers in the control and AD-MCI groups but not the AD-dementia group. Our findings suggest that, with higher levels of Klotho, progression along the AD continuum is less likely and that the association of *KL-VS* heterozygosity status with higher Klotho levels is abolished in the final stages of the disease.

A previous study showed a negative correlation of CSF Klotho levels with age in a combined cohort of controls and patients with AD.^15^ We confirmed this finding for the control group, but in the AD group, this age correlation was lost. This finding indicates that the decrease of CSF Klotho levels occurs at a younger age in AD patients. Klotho expression is regulated by promoter methylation, and increased methylation with age results in downregulated expression of Klotho.^30,31^ Thus, our results suggest that this epigenetic mechanism might be dysregulated early in the pathogenesis of AD. An increased Klotho promoter methylation level has already been described in several malignant neoplasms, and epigenetic silencing of Klotho expression was associated with poor prognosis.^31,32,33,34^

In this study, we demonstrate a significant positive association of CSF Klotho levels with CSF Aβ42 levels independent of clinical, *KL-VS* heterozygosity, or *APOE4* status, suggesting that CSF Klotho levels are associated with amyloid burden among all individuals. No study has explored the association of CSF Klotho levels with amyloid burden, to our knowledge. Several studies have shown that the *KL-VS* heterozygosity haplotype is associated with reduced amyloid burden in controls carrying *APOE4*,^10,11,12^ whereas 1 study could not confirm these results.^35^ We found no significant association between *KL-VS* heterozygosity and CSF Aβ42 levels, but this finding should be interpreted with caution owing to our somewhat limited sample size. This was evident in a recent study that had to double the sample size to identify a significant association between *KL-VS* heterozygosity and amyloid burden.^13^ Together, the association of the CSF Klotho protein levels, but not the genetic variant *KL-VS* heterozygosity, with levels of amyloid indicates that the results are mediated more robustly by the protein and not indirectly by the genotype that is correlated with higher protein levels. Thus, an association between CSF Klotho levels and amyloid burden is detectable, even in a cohort with low sample size. It is also suggested that *KL-VS* heterozygosity not only increases the concentration of Klotho but also changes its function, making the possible protective effect of CSF Klotho even more potent.^36^

A possible protective effect of *KL-VS* heterozygosity on tau accumulation has been explored to a lesser extent,^13,14^ and we are the first, to our knowledge, to report an association between CSF Klotho levels and CSF T-tau and P-tau levels in a clinical cohort with AD. This association was independent of clinical stage and *KL-VS* heterozygosity or *APOE4* status. All our controls were amyloid negative, which indicates that higher levels of CSF Klotho may be associated with less formation of neurofibrillary tangles and may be associated with neuroprotective mechanisms in all individuals, independent of clinical or amyloid status. We could not find a significant association between the *KL-VS* heterozygosity haplotype and T-tau or P-tau levels, nor in the stratified analysis for *APOE4* carriers and noncarriers, in contrast to 2 recent studies^13,14^ that demonstrated a protective association of *KL-VS* heterozygosity with tau burden. The deviation with our study might again be explained by low sample sizes in the groups when stratified by the *KL-VS* haplotype. The explanation as to how the Klotho level is associated with tau pathology is still unclear, but it has been linked to several biological processes, including calcium signaling,^37^ growth factor functions,^38^ insulin regulation,^3^ and reactive oxygen species regulation, thereby decreasing neuronal damage.^39,40^ It has also been speculated whether Klotho’s involvement in autophagy might be associated with the clearance of AD-related proteins.^41,42^

The significant association of CSF Klotho levels with lower tau burden suggests a potential association of Klotho levels with cognitive function. There was a significant but weak positive association between CSF Klotho levels and MMSE score in the AD group but not the control group, although the smaller variation in MMSE scores in the control group might have been associated with the result. This finding indicates that lower levels of CSF Klotho could be associated with greater cognitive decline among patients with AD. Two previous studies have demonstrated a similar positive correlation, but in heterogenous cohorts of patients with AD and other types of dementia, as well as controls.^15,43^ Cognitive decline and gray matter atrophy are closely associated with the progressive development of neurofibrillary tangles in the presence of amyloid pathology and are more likely associated with AD disease progression than amyloid burden.^44,45,46^ Taking this into account, the correlation between CSF Klotho levels and MMSE score in the AD group in our study might be mediated by the association of CSF Klotho levels and tau pathology.

There was a weak correlation between CSF and plasma Klotho levels in our study population, but we could not confirm that plasma Klotho levels were associated with clinical stages of AD or amyloid burden, as seen with CSF Klotho levels. Plasma Klotho levels were negatively associated with tau burden independent of clinical stage, indicating that higher levels of Klotho in blood may be associated with less neurodegeneration in general, independent of AD-specific amyloid pathology. Studies have also shown an association between lower levels of plasma Klotho and lower MMSE scores in older age and a higher risk for vascular dementia^17,47^ but not with an increased risk of AD.^48^ As with CSF Klotho levels, plasma Klotho levels were negatively correlated with age in the control group but not the AD group, suggesting a tissue-independent decrease in Klotho expression at a younger age among individuals with AD. The kidney produces Klotho for the blood, and the choroid plexus in the brain produces Klotho for the CSF.^49^ It is therefore natural to assume that CSF Klotho has a greater association with brain function.

### Limitations

This case-control study has some limitations. First, the need for CSF collection led to the low sample size, especially in the AD-dementia group. Second, the cross-sectional design did not allow any causal interpretations. Third, all controls had Aβ42 CSF levels below the pathologic threshold, eliminating the possibility to study the association of Klotho levels with preclinical AD.

## Conclusions

Our findings suggest that the Klotho protein, but not the *KL-VS* heterozygosity haplotype, differs in clinical stages of AD and is associated with cognitive decline and amyloid and tau burden. This may be explained by an accelerated decrease in Klotho during aging in patients with AD. The mechanisms causing the favorable effects of Klotho and methods for increasing the level of Klotho should be further explored.

## Notes

Supplement. **eFigure 1.** CSF Klotho by KL-VS Heterozygosity in the Clinical Groups **eFigure 2.** Correlation Between CSF and Plasma Levels of Klotho **eFigure 3.** Scatterplot of Plasma Klotho and Age in the Clinical Groups **eTable 1.** Associations Between CSF Klotho and Core CSF AD Biomarkers in Clinical Groups **eTable 2.** Associations Between CSF Klotho and AD Core CSF Biomarkers in Subgroups Based on *APOE4*+ and *KL-VS^HET+^* Status **eTable 3.** Associations Between Plasma Klotho and Core CSF AD Biomarkers
