# Supplementary material for: Association of Klotho Protein Levels and KL-VS Heterozygosity With Alzheimer Disease and Amyloid and Tau Burden
Source: JAMA Netw Open. 2022 Nov 22;5(11):e2243232. doi: 10.1001/jamanetworkopen.2022.43232 (PMC9682425; doi:10.1001/jamanetworkopen.2022.43232)
Supplement: Supplement. — eFigure 1. CSF Klotho by KL-VS Heterozygosity in the Clinical Groups eFigure 2. Correlation Between CSF and Plasma Levels of Klotho eFigure 3. Scatterplot of Plasma Klotho and Age in the Clinical Groups eTable 1. Associations Between CSF Klotho and Core CSF AD Biomarkers in Clinical Groups eTable 2. Associations Between CSF Klotho and AD Core CSF Biomarkers in Subgroups Based on APOE4+ and KL-VSHET+ Status eTable 3. Associations Between Plasma Klotho and Core CSF AD Biomarkers [file jamanetwopen-e2243232-s001.pdf]

Supplementary Online Content

Grøntvedt GR, Sando SB, Lauridsen C, et al. Association of Klotho protein levels and *KL-VS* heterozygosity with Alzheimer disease and amyloid and tau burden. *JAMA Netw Open.* 2022;5(11):e2243232.
doi:10.1001/jamanetworkopen.2022.43232

eFigure 1. CSF Klotho by KL-VS Heterozygosity in the Clinical Groups
eFigure 2. Correlation Between CSF and Plasma Levels of Klotho
eFigure 3. Scatterplot of Plasma Klotho and Age in the Clinical Groups
eTable 1. Associations Between CSF Klotho and Core CSF AD Biomarkers in Clinical Groups
eTable 2. Associations Between CSF Klotho and AD Core CSF Biomarkers in Subgroups Based on *APOE4*+ and *KL-VS*$^{HET+}$ Status
eTable 3. Associations Between Plasma Klotho and Core CSF AD Biomarkers

This supplementary material has been provided by the authors to give readers additional information about their work.

© 2022 Grøntvedt GR et al. *JAMA Network Open.*

eFigure 1. CSF Klotho by KL-VS Heterozygosity in the Clinical Groups[a]

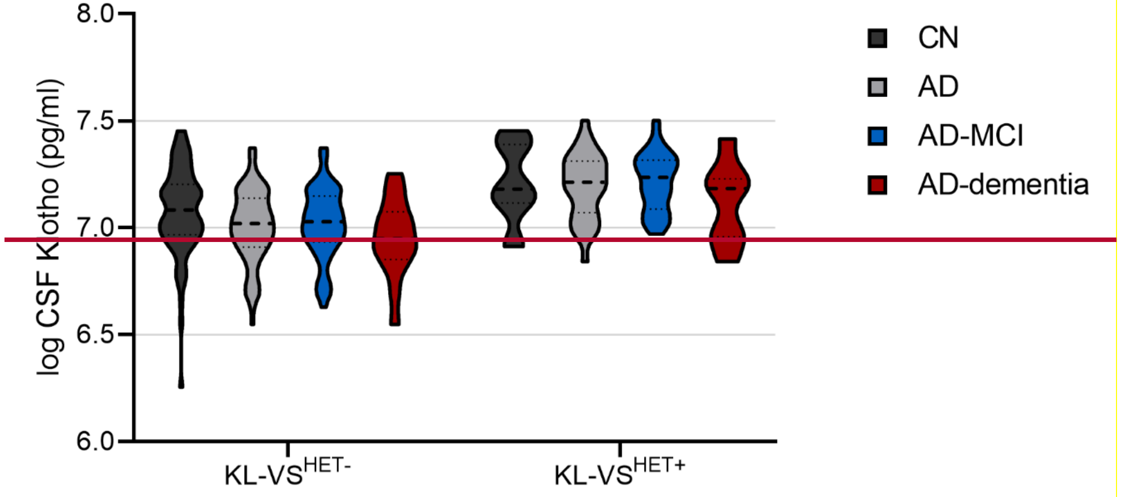

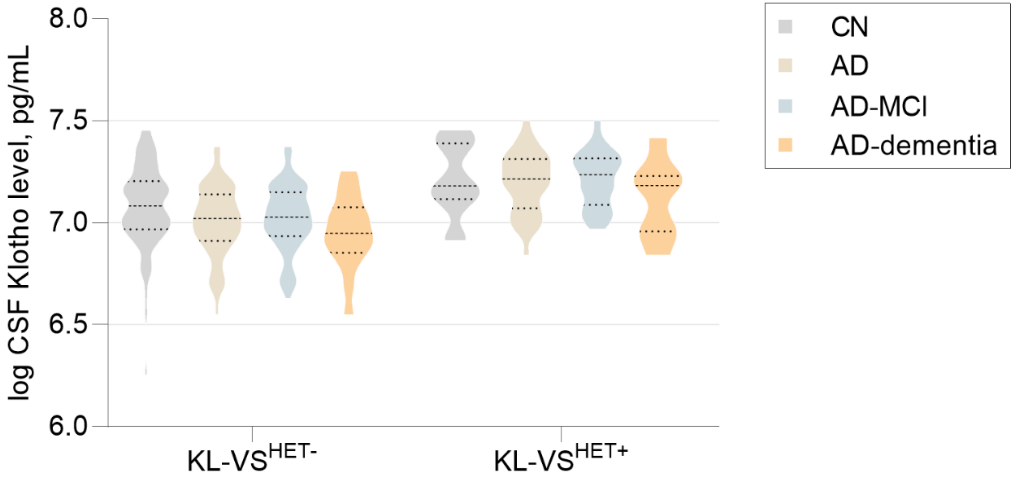

Abbreviations: AD-MCI, mild cognitive impairment due to Alzheimer´s disease. AD-dementia, dementia due to Alzheimer´s disease. CN, healthy controls. AD group, Alzheimer´s disease group. *KL-VS,* haplotype consisting of two missense variants in the *Klotho* gene. KL-VS HET+, heterozygosity for *KL-VS*. KL-VS HET-, no heterozygosity for *KL-VS*. CSF, cerebrospinal fluid.
[a] This violin plot shows the concentrations of Klotho in CSF (pg/mL) based on *KL-VS* HET+ status in different clinical groups.

© 2022 Grøntvedt GR et al. *JAMA Network Open.*

eFigure 2. Correlation Between CSF and Plasma Levels of Klotho[a]

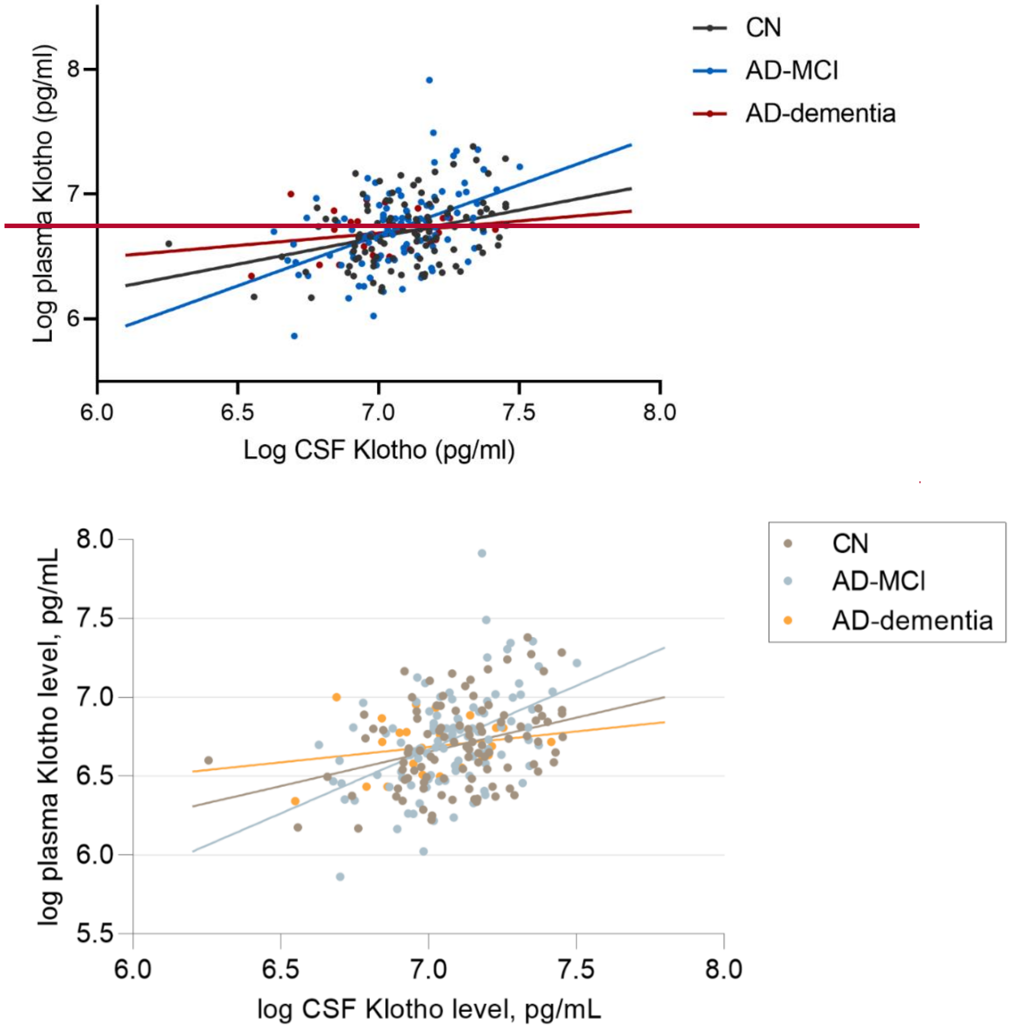

Abbreviations: AD-MCI, mild cognitive impairment due to Alzheimer´s disease. AD-dementia, dementia due to Alzheimer´s disease. CN, healthy controls. CSF, cerebrospinal fluid.
[a]This scatterplot shows the correlation between Klotho in CSF (pg/mL) and Klotho in plasma (pg/mL) in clinical groups measured by commercially available Human soluble α-Klotho Assay ELISA kit.

© 2022 Grøntvedt GR et al. *JAMA Network Open.*

eFigure 3. Scatterplot of Plasma Klotho and Age in the Clinical Groups[a]

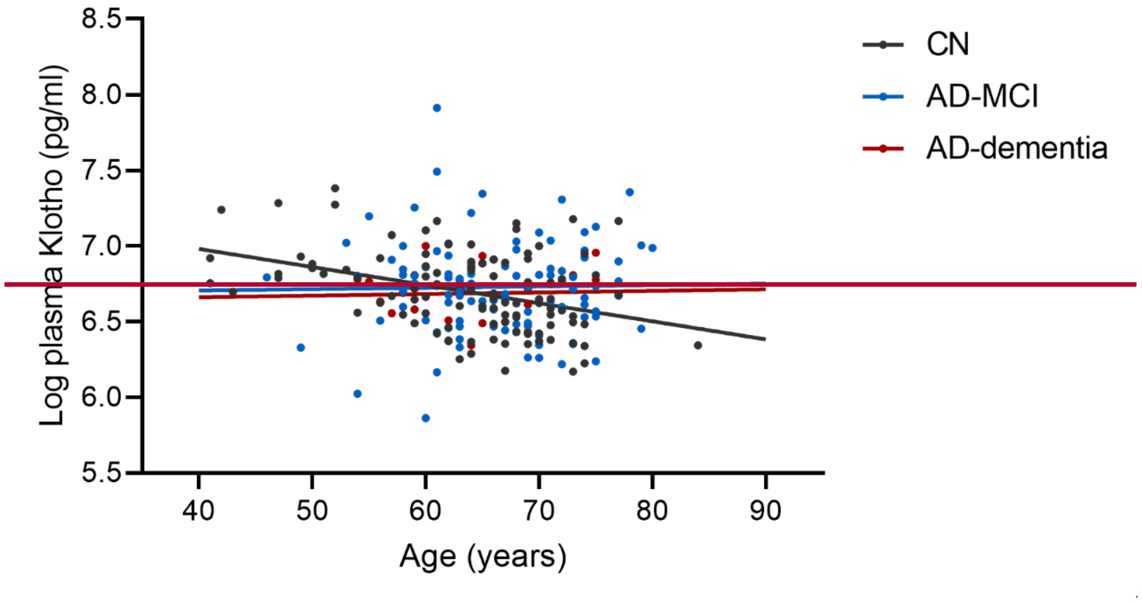

© 2022 Grøntvedt GR et al. *JAMA Network Open.*

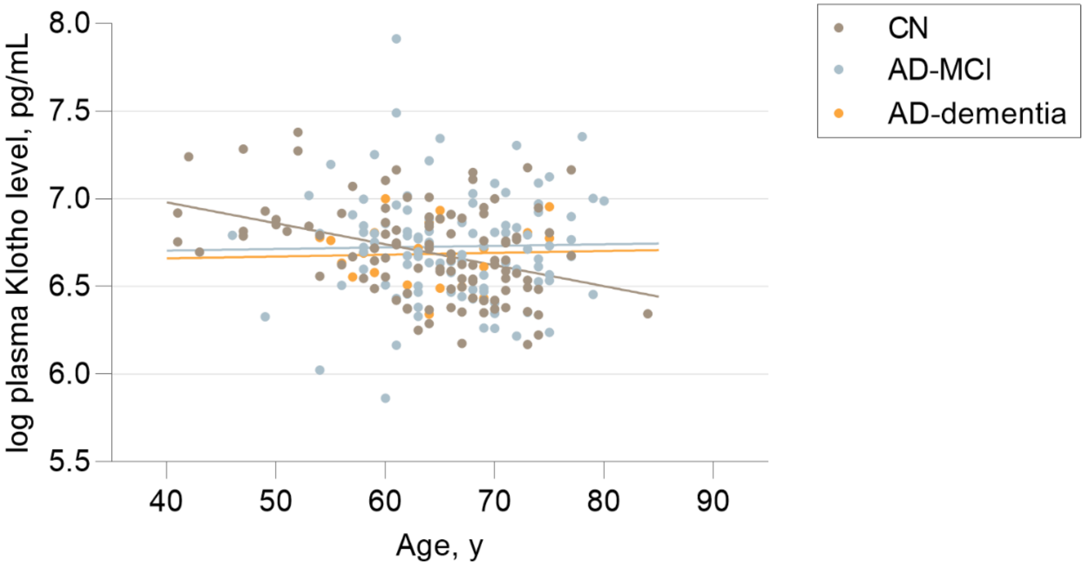

Abbreviations: AD-MCI, mild cognitive impairment due to Alzheimer´s disease. AD-dementia, dementia due to Alzheimer´s disease. CN, healthy controls.
[a] This scatterplot shows logarithmic transformed concentrations values of Klotho in plasma and age in different clinical groups.

© 2022 Grøntvedt GR et al. *JAMA Network Open.*

eTable 1. Associations Between CSF Klotho and Core CSF AD Biomarkers in Clinical Groups[d]

		Beta coefficient	95% confidence interval
**CSF AB42[a]**	CN	0.104	-0.139 – 0.347
	AD	0.099	-0.142 – 0.341
**CSF T-tau[b]**	CN	-0.360	-0.871 – 0.151
	AD	-0.723	-1.295 – -0.152
**CSF P-tau[c]**	CN	-0.233	-0.644 – 0.178
	AD	-0.556	-1.014 – -0.099

Abbreviations: CN=control group. AD group= Alzheimer´s disease group. CSF=cerebrospinal fluid. Aβ42=amyloid beta 42. T-tau= total tau. P-tau= phosphorylated tau.
[a] No significant difference between clinical groups, p=0.260
[b] No significant difference between clinical groups, p=0.399
[c] No significant difference between clinical groups, p=0.349
[d] This table shows the associations between CSF klotho and CSF using univariable linear regression.

© 2022 Grøntvedt GR et al. *JAMA Network Open.*

eTable 2. Associations Between CSF Klotho and AD Core CSF Biomarkers in Subgroups Based on *APOE4*+ and *KL-VS*$^{HET+}$ Status[g]

Subgroup	CSF Aβ42			CSF T-tau		CSF P-tau	
	**N**	**Beta Coefficient**	**95%CI**	**Beta Coefficient**	**95%C.**	**Beta Coefficient**	**95%CI**
**CN and *APOE4-***	37	0.091	-0.224 - 0.407 [a]	-0.554	-0.318 0.211 [b]	-0.385	-1.036 - 0.265 [c]
**CN and *APOE4+***	24	0.078	-0.338 - 0.794	-0.169	-0.950 - 0.613	-0.061	-0.651 – 0.530
**AD and *APOE4-***	27	-0.243	-0.594 - 0.108	-0.865	-2.394 - 0.663	-0.062	-0.213 – 1.090
**AD and *APOE4+***	97	0.228	-0.065 – 0.521	-0.700	-1.284 - -0.116	-0.748	-1.236 - -0.260
**CN and *KLVS*$^{HET-}$**	50	0.100	-0.155 – 0.355 [d]	-0.448	-0.024 – 0.128 [e]	-0.337	-0.787 – 0.113 [f]
**CN and *KLVS*$^{HET+}$**	13	0.497	-0.247 – 1.241	-0.302	-1.780 – 1.176	-0.015	-1.287 – 1.267
**AD and *KLVS*$^{HET-}$**	90	0.090	-0.232 – 0.412	-0.811	-1.515 - -0.108	-0.695	-1.279 - -0.110
**AD and *KLVS*$^{HET+}$**	36	0.308	-0.235 – 0.850	-0.178	-1.722 – 1.366	-0.512	-1.652 – 0.628

Abbreviatons: CN= control group. AD= Alzheimer´s disease group. *APOE4+= Apolipoprotein E4 carrier. APOE4-= Apolipoprotein E4 non- carrier. KLVS*$^{HET+}$ = Heterozygosity for *KL-VS. KLVS*$^{HET-}$*= no heterozygosity for KL-VS.* Aβ42=amyloid beta 42. T-tau= total tau. P-tau= phosphorylated tau.

[a] No significant differences between subgroups, p=0.337

[b] No significant differences between subgroups, p=0.706

[c] No significant differences between subgroups, p=0.297

[d] No significant differences between subgroups, p=0.683

[e] No significant differences between subgroups, p=0.774

[f] No significant differences between subgroups, p=0.694

[g]This table shows the associations between CSF Klotho and core CSF AD biomarkers in subgroups based on *APOE4*+ and *KL-VS*$^{HET+}$ status using univariable linear regression.

© 2022 Grøntvedt GR et al. *JAMA Network Open.*

eTable 3. Associations Between Plasma Klotho and Core CSF AD Biomarkers[a]

Entire cohort

CSF Aβ42: p=0.133.	Beta coefficient = 0-176	95%CI -0.054 - -0.406
CSF T-tau: p=0.015,	Beta coefficient = -0.398	95%CI -0.719 - -0.077
CSF P-tau: p=0.042,	Beta coefficient = -0.268	95%CI -0.526 - -0.010.

No difference in this association between the CN group and the AD group:

CSF Aβ42 p=0.523	CSF T-tau p=0.427	CSF P-tau p=0.503

***No difference in this association between the CN group and the AD group based on KL-VS^{HET+} status*:**

CSF Aβ42 p=0.813	CSF T-tau, p=0.840	CSF P-tau p=0.907

***No difference in this association between the CN group and the AD group based on APOE4+ status*:**

CSF Aβ42 p=0.730	CSF T-taup=0.518	CSF P-tau p=0.480

Abbreviations: CN= control group. AD= Alzheimer´s disease group. *APOE4+= Apolipoprotein E4 carrier. KLVS^{HET+}* = Heterozygosity for *KL-VS.* Aβ42=amyloid beta 42. T-tau= total tau. P-tau= phosphorylated tau.

[a]This Table shows the associations between plasma Klotho and core CSF AD biomarkers in the entire cohort and further stratified by clinical groups, *APOE4*+ - and *KL-VS^{HET+}* status using univariable linear regression.

© 2022 Grøntvedt GR et al. *JAMA Network Open.*
